# Impact of Sequence Variation in the UL128 Locus on Production of Human Cytomegalovirus in Fibroblast and Epithelial Cells

**DOI:** 10.1128/JVI.01546-13

**Published:** 2013-10

**Authors:** Isa Murrell, Peter Tomasec, Gavin S. Wilkie, Derrick J. Dargan, Andrew J. Davison, Richard J. Stanton

**Affiliations:** Institute of Infection and Immunity, School of Medicine, Cardiff University, Cardiff, United Kingdoma; MRC–University of Glasgow Centre for Virus Research, Glasgow, United Kingdomb

## Abstract

The human cytomegalovirus (HCMV) virion envelope contains a complex consisting of glycoproteins gH and gL plus proteins encoded by the UL128 locus (UL128L): pUL128, pUL130, and pUL131A. UL128L is necessary for efficient infection of myeloid, epithelial, and endothelial cells but limits replication in fibroblasts. Consequently, disrupting mutations in UL128L are rapidly selected when clinical isolates are cultured in fibroblasts. In contrast, bacterial artificial chromosome (BAC)-cloned strains TB40-BAC4, FIX, and TR do not contain overt disruptions in UL128L, yet no virus reconstituted from them has been reported to acquire mutations in UL128L *in vitro*. We performed BAC mutagenesis and reconstitution experiments to test the hypothesis that these strains contain subtle mutations in UL128L that were acquired during passage prior to BAC cloning. Compared to strain Merlin containing wild-type UL128L, all three strains produced higher yields of cell-free virus. Moreover, TB40-BAC4 and FIX spread cell to cell more rapidly than wild-type Merlin in fibroblasts but more slowly in epithelial cells. The differential growth properties of TB40-BAC4 and FIX (but not TR) were mapped to single-nucleotide substitutions in UL128L. The substitution in TB40-BAC4 reduced the splicing efficiency of UL128, and that in FIX resulted in an amino acid substitution in UL130. Introduction of these substitutions into Merlin dramatically increased yields of cell-free virus and increased cell-to-cell spread in fibroblasts but reduced the abundance of pUL128 in the virion and the efficiency of epithelial cell infection. These substitutions appear to represent mutations in UL128L that permit virus to be propagated in fibroblasts while retaining epithelial cell tropism.

## INTRODUCTION

Human cytomegalovirus (HCMV) is ubiquitous throughout populations worldwide and represents a significant public health challenge in both developed and developing countries (1). Like other herpesviruses, HCMV establishes life-long persistent infections with periodic episodes of reactivation that require constant immunosurveillance. Productive infection is commonly asymptomatic in an immunocompetent host, but the virus remains a leading infectious cause of congenital malformation and is responsible for a broad spectrum of pathological consequences in immunocompromised (e.g., AIDS patients) or immunosuppressed individuals (e.g., transplant recipients). HCMV displays tropism for a broad range of cell types and tissues *in vivo*, with disease being associated with most major organs. For example, initial infection occurs in mucosal epithelial tissues, infection of endothelial tissues can result in transfer of virus to solid organs and leukocytes for dissemination, and CD34^+^ bone marrow progenitor cells and monocytes are sites of latency (1–3). Thus, studies of HCMV pathogenesis require the use of virus with the ability to infect a wide range of cell types *in vitro*. However, there are significant challenges associated with *in vitro* propagation of virus that exhibits the broad tropism characteristic of clinical virus.

Classically, three distinct virion envelope glycoprotein complexes, designated gCI, gCII, and gCIII, have been implicated in recognition and uptake of HCMV by the cell. gCI, composed of glycoprotein gB, and gCII, composed of glycoproteins gM and gN, mediate the initial attachment of virions. Both are capable of binding to heparin sulfate proteoglycans (4–7), while gB is also capable of binding to integrins, epidermal growth factor receptor (EGFR), and platelet-derived growth factor receptor α (PDGFRα). All of these molecules have been reported to be important for virus entry (8–11), although the roles of EGFR and PDGFRα have been disputed (12, 13). Following initial binding, fusion with cellular membranes is orchestrated by gB and gCIII, which is formed of glycoproteins gH, gL, and gO (14–18). More recently it has become apparent that gH and gL also form a second glycoprotein complex, and that infection of different cell types occurs by different mechanisms involving these two different complexes.

Infection of fibroblasts occurs by direct fusion of the virion envelope with the plasma membrane, whereas in epithelial, endothelial, and myeloid cells, membrane fusion takes place in vesicles following internalization by endocytosis or micropinocytosis (19–22). gH/gL/gO is required for infection, virion maturation, egress, and cell-to-cell spread in fibroblasts, as well as for infection of epithelial and endothelial cells (6, 23, 24). A second complex, gH/gL/UL128L, is formed by gH/gL along with the products of the UL128 locus (UL128L), pUL128, pUL130, and pUL131A. gH/gL/UL128L is required for efficient infection and cell-to-cell spread in epithelial, endothelial, and myeloid cells (22, 25–35), either by binding to cell surface receptors (22, 27, 28) or by promoting nuclear translocation of virions (21, 32, 36). Infection of fibroblasts does not require gH/gL/UL128L; in fact, virus containing gH/gL/UL128L displays reduced cell-to-cell spread and cell-free release in fibroblasts *in vitro* (37, 38). As a result, there is considerable selection pressure against UL128L in this cell type. Thus, routine isolation of HCMV strains from clinical material in fibroblasts is associated with rapid acquisition of disabling mutations in UL128L, which are usually apparent as frameshifts caused by insertion or deletion of one or more nucleotides, in-frame termination codons caused by single-nucleotide substitutions, or deletions (37–44). This results in the generation of laboratory-adapted viruses that display efficient growth in fibroblasts but limited growth in other cell types.

To provide a genetically stable source of HCMV, the genome can be cloned into a bacterial artificial chromosome (BAC) and virus recovered by transfection (38, 45–47). However, HCMV is invariably subjected to some degree of passaging *in vitro* prior to BAC cloning, and as a result, BAC-cloned strains exhibit various degrees of *in vitro* adaptation. We have previously described the cloning of the complete HCMV strain Merlin genome into a self-excising BAC following five passages in fibroblasts (38). *In vitro*-acquired mutations were identified by reference to the original clinical material and repaired, with the resulting BAC having the genetic competence of wild-type virus. However, as with clinical HCMV strains, the presence of wild-type UL128L in reconstituted virus results in the production of very low titers of cell-free virus *in vitro*, and the virus is prone to mutation when passaged in fibroblasts (38).

Several HCMV strains in addition to Merlin have been BAC cloned. These include TB40-BAC4, which was cloned from a mixed population of TB40/E following five passages in fibroblasts and 22 passages in endothelial cells (48), FIX, which was derived from strain VR1814 following 46 passages in fibroblasts (49, 50), and TR, which was isolated from an ocular swab from an AIDS patient (51, 52). Strains TB40-BAC4, FIX, and TR were cloned by replacing sequences in one region of the genome (at the left end of U_s_) with a nonexcising BAC vector. UL128L in each BAC is apparently intact, and reconstituted virus is able to infect epithelial and endothelial cells. However, unlike strains isolated from clinical material, there are no reports of viruses reconstituted from these BACs acquiring mutations in UL128L during passage *in vitro*. This raised the hypothesis that these viruses contain subtle mutations in UL128L that were acquired during passage of the clinical isolates prior to BAC cloning. Indeed, our study identifies single-nucleotide substitutions in strains TB40-BAC4 and FIX that impact UL128 and UL130, respectively. Introduction of these substitutions into wild-type UL128L in strain Merlin dramatically increased yields of cell-free virus and increased cell-cell spread in fibroblasts but reduced both the abundance of pUL128 in the virion and the efficiency of infection in epithelial cells.

## MATERIALS AND METHODS

### Cells and viruses.

Primary human fetal foreskin fibroblast (HFFF) cells and human telomerase reverse transcriptase (hTERT)-immortalized retinal pigmented epithelial (RPE-1) cells were grown in Dulbecco's modified Eagle medium (DMEM) (Life Technologies) supplemented with fetal bovine serum (10%, vol/vol), penicillin (500 U/ml), and streptomycin (500 μg/ml) at 37°C in 5% CO_2_. Two variants of the Merlin BAC were used and have been described previously (38). Merlin-UL128L^mut^ (previously called pAL1158) contains a premature stop codon in UL128, while Merlin-UL128L^wt^ (previously called pAL1160) contains wild-type UL128. Both BACs contain a frameshift in RL13 and an internal ribosomal entry site (IRES) followed by enhanced green fluorescent protein (EGFP) after UL122. TB40-BAC4 was kindly donated by Christian Sinzger (48), FIX by Gabi Hahn (50), and TR by Jay Nelson (51). For these BACs, recombineering was used to insert an IRES followed by EGFP after UL122 as described previously (38). HCMV strain 3301 DNA had been extracted previously from the urine of a congenitally infected infant (40).

Infections were performed at 37°C for 2 h on a rocker, followed by removal of the inoculum and addition of fresh medium (53). In titrations and assays to investigate cell-to-cell spread, supernatant-driven spread was limited by use of a 1% Avicel semisolid overlay (54). After incubation for 14 days (for fibroblasts) or 21 days (for epithelial cells), the overlay was removed and cells were washed in phosphate-buffered saline (PBS). Unless otherwise stated, quantitation of cell-free virus produced during infection of both fibroblast and epithelial cells was performed using HFFF, since this is the only cell type in which all viruses could infect and spread. Plaques were identified based on EGFP expression and imaged using an ORCA-ER camera and Leica DMIRBE microscope. Plaque sizes were determined using OpenLab 3 software.

### Preparation of BACs.

Stocks of each BAC-cloned genome were prepared using a Nucleobond plasmid purification kit (Macherey-Nagel) according to the manufacturer's instructions. The concentration of purified plasmid DNA was determined by use of an ND1000 spectrophotometer (Nanodrop).

### Transfections.

BACs were transfected into HFFF cells by electroporation using a Nucleofector (Amaxa) and basic fibroblast kit (Lonza) and program T-16 according to the manufacturer's instructions. On occasions when the monolayer formed was less than 70% confluent on the following day, additional cells were added. The number of plaques formed following electroporation varied only marginally, ranging from 20 to 35 per transfection. RPE-1 cells were transfected using Effectene (Qiagen) according to the manufacturer's instructions. The number of plaques formed varied only minimally, with 50 to 70 plaques generated per transfection. These variations were not observed to have a major impact on the relative rate of virus spread through the monolayer in experimental repeats.

### FACS analysis.

At weekly time points posttransfection, infected RPE-1 cultures were trypsinized and reseeded into fresh flasks. An aliquot of cells was kept for fluorescence-activated cell sorter (FACS) analysis using an Accuri C6 and CFlow software for the detection of EGFP^+^ cells.

### Recombineering.

All recombineering was performed as described previously (38, 55, 56), using E. coli SW102 cells containing the BAC to be modified. A selectable *amp*/*sacB*/*lacZ* cassette was PCR amplified and inserted into the region to be modified, followed by positive selection for expression of ampicillin resistance on medium supplemented with ampicillin (50 μg/ml). In a second round of recombineering, the selection cassette was swapped with the DNA sequence to be inserted, followed by negative selection on medium supplemented with sucrose (5%, wt/vol) to select against *sacB* expression and 5-bromo-4-chloro-3-indolyl-β-*d*-galactopyranoside (X-Gal) and isopropyl β-*d*-1-thiogalactopyranoside (IPTG) to identify white colonies lacking *lacZ* expression. Amplification of the selectable cassette was performed using the Expand HiFi system (Roche) under the following conditions: 95°C for 2 min; 10 cycles at 95°C for 30 s, 55°C for 30 s, and 68°C for 4.5 min; 25 cycles at 95°C for 30 s, 55°C for 30 s, and 68°C for 4.5 min; and 68°C for 15 min. Primer pairs were designed with approximately 20 bp of identity to the selectable cassette at each 3′ end and approximately 80 bp of identity to sequences adjacent to the insertion site at the 5′ end. In the primer sequences shown below, regions identical to sequences immediately up- and downstream from the insertion site are underlined. Primers were designed to cover regions with 100% identity in all strains.

### Insertion of UL128L sequences into the Merlin genome.

For insertion of the complete UL128L from strains TR, TB40-BAC4, FIX, and 3301 in place of the wild-type Merlin UL128L, the *amp*/*sacB*/*lacZ* cassette was amplified using primers SacBR-131A (CAG TCT GCA ACA TGC GGC TGT GTC GGG TGT GGC TGT CTG TTT GTC TGT GCG CCG TGG TGC TGG GTC AGT GCC AGC GGG ACT GAG GTT CTT ATG GCT CTT G) and SacBF-128 (ATC CAG CCG TTT GTG TTT CTT AAC GCT CTC CAG GTA CTG ATC CAG GCC CAC GAT CCG GGT TAT CTT GTC GTA TTC CAG CCT GTG ACG GAA GAT CAC TTC G). UL128L from each strain was amplified using a Phusion high-fidelity kit (New England BioLabs) and primers UL128LF (GCG TAT TTC GGA CAA ACA CAC A) and UL128LR (CGC ATG TTG CAG ACT GAG AAA GA). PCR was performed under the following conditions: 98°C for 1 min; 35 cycles at 98°C for 30 s, 55°C for 30 s, and 72°C for 1 min; and 72°C for 10 min.

### Insertion of the unique TB40-BAC4 nucleotide into the Merlin genome.

The *amp*/*sacB*/*lacZ* selection cassette was amplified using primers SacBF (GGT GGT GAC GAT CCC GCG AAT CTC AGC CGT TTT CTC GGG ACT GTA GCA GAC TTC GCC GTC CGG ACA CCG CAG CCT GTG CCT GTG ACG GAA GAT CAC TTC G) and SacBR (CTG GAT CTG TCT CTC GAC GTT TCT GAT AGC CAT GTT CCA TCG ACG ATC CTC GGG AAT GCC AGA GTA GAT TTT CAT GAA TCT GAG GTT CTT ATG GCT CTT G). The following oligonucleotide was used to insert the TB40-BAC4 UL128 nucleotide (underlined): GTT TTC TCG GGA CTG TAG CAG ACT TCG CCG TCC GGA CAC CGC AGC CTG TTG ATT CAT GAA AAT CTA CTC TGG CAT TCC CGA GGA TCG TCG ATG GAA CAT G.

### Insertion of unique FIX UL130 nucleotide into the Merlin genome.

The *amp*/*sacB*/*lacZ* selection cassette was amplified using primers SacBF-FIX (GTC TGG CCT TCC CGG TTG TAC AGC AGA TAC AGG GTC TCG TTG CGA CAC TCG GGA CCC GTT GAT ACC CGC TGG AAC CCC CCT GTG ACG GAA GAT CAC TTC G) and SacBR-FIX (TAT TCC AAA CCG CAT GAC GCG GCG ACG TTT TAC TGT CCT TTT CTC TAT CCC TCG CCC CCA CGA TCC CCC TTG CAA TTC CCT GAG GTT CTT ATG GCT CTT G). The following oligonucleotide was used to insert the FIX UL130 nucleotide (underlined): AGG GTC TCG TTG CGA CAC TCG GGA CCC GTT GAT ACC CGC TGG AAC CCC GAT AAT TGC AAG GGG GAT CGT GGG GGC GAG GGA TAG AGA AAA GGA CAG TAA A.

### Sanger DNA sequencing.

UL128L was amplified by PCR using primers UL128LF (GCG TAT TTC GGA CAA ACA CAC A) and UL128LR (CGC ATG TTG CAG ACT GAG AAA GA). UL128L was amplified from all viruses during time courses (e.g., from infected cell culture supernatants) to determine whether it retained its original sequence. The Advantage II PCR system (Clontech) was used according to the manufacturer's instructions under the following conditions: 95° for 10 min; 35 cycles at 95°C for 30 s, 55°C for 30 s, and 68°C for 3 min; and 68°C for 10 min. When UL128L was amplified from a BAC, the HiFi expand PCR system (Roche) was used according to the manufacturer's instructions under the following conditions: 94°C for 2 min; 30 cycles at 94°C for 15 s, 68°C for 30 s, and 72°C for 2 min; and 72°C for 7 min. PCR products were purified from agarose gels by using an Illustra GFX PCR DNA gel band purification kit (GE Healthcare) and then sequenced (EurofinsMWG) using the primers CGG ATT GTA GTT GCA GCT CG, TCT GGT TAT TGG CCT CGG TG, TCT TCC AAT ATC GCC ATC TC, and GCG CAC AGA AGC AGG CAG. Sequence alignments, BLAST searches, and other analyses were performed by using CLC MAIN 6 software.

### Generation of cDNA libraries of UL128 transcripts.

Total cell RNA samples from HFFF cultures infected at a multiplicity of infection (MOI) of 3 were extracted 72 h postinfection by using an RNeasy plus universal kit (Qiagen) according to the manufacturer's instructions. cDNAs were generated using a NanoScript Precision reverse transcriptase kit (Primer Design). cDNAs were then amplified using primers UL128F (CATAAACGTCAACCACCC) and UL128R (CACTGCAGCATATAGCCC) with the HiFi expand PCR system (Roche) under the conditions stated above.

### Bioinformatics analysis.

Analysis of UL128 splice sites was carried out by using NNsplice v. 0.9 (57), NetGene2 (58), SplicePort (59), and Human Splicing Finder v. 2.4.1 (60). Protein structure prediction was performed using Phyre 2 (61).

### Preparation of virus stocks.

Where feasible, virus stocks were generated in RPE-1 cells to prevent the selection of mutations in UL128L. Merlin strains mutated in UL128L and FIX were grown in HFFF cells owing to their inability to spread productively in RPE-1 cells. Supernatant from infected cell cultures was collected and centrifuged at 470 × *g* for 5 min at room temperature to pellet cellular debris. Cleared supernatants were centrifuged at 30,000 × *g* for 2 h at 20°C. Pelleted virions were resuspended in DMEM containing 10% fetal bovine serum (FBS) and stored at −80°C.

### Gradient purification of virions.

Virions were purified from noninfectious enveloped particles, dense bodies, and cellular debris by ultracentrifugation through glycerol-tartrate positive-density, negative-viscosity gradients, as described previously (62, 63). The gradients were centrifuged at 90,465.7 × *g* for 45 min at 20°C. Virions were recovered by piercing the tubes using a 20-gauge needle and syringe, diluted in 0.04 M sodium-phosphate buffer (pH 7.4), and pelleted by centrifugation at 90,465.7 × *g* for 1 h at 20°C. The virion pellet was resuspended in a medium suitable for downstream applications (see below).

### Western blot analysis.

Purified virions were resuspended in Nu-Page LDS sample buffer, and proteins were separated by using a Nu-page Tris-acetate gel system (Invitrogen) according to the manufacturer's instructions. Electrophoresed proteins were subsequently transferred to Hybond-P polyvinylidene difluoride (PVDF) membranes (GE Healthcare) by semidry transfer at 10 V for 1 h using carbonate transfer buffer. The membranes were incubated for 1 h at room temperature in blocking buffer (PBS containing 0.1% Tween 20 [PBST] plus 5% [wt/vol] fat-free milk). They were then incubated with primary antibody for 1 h at room temperature, washed three times with PBST, and incubated with secondary antibody for 1 h at room temperature. Antibody was detected by SuperSignal West Pico (Thermo) using an AutoChemi imaging system and Labworks software (UVP Bioimaging). Primary antibodies were mouse-anti pUL128 antibody SURN, provided by Giueseppe Gerna (1:100), and mouse anti-gB (1:4,000; Abcam). The secondary antibody was goat anti-mouse horseradish peroxidase (HRP; 1:1,000; GE Healthcare).

## RESULTS

### Growth properties of HCMV strains FIX, Merlin, TB40-BAC4, and TR in fibroblast cells.

Clinical isolates of HCMV consistently replicate inefficiently in fibroblast cultures until mutations arise and are selected, first in RL13 and then in UL128L (37, 38). A defect in any of UL128, UL130, or UL131A suppresses formation of the pentameric glycoprotein complex (gH/gL/UL128L) in the virion (37). To establish the growth characteristics of virus lacking or containing gH/gL/UL128L, viruses derived from two Merlin BAC variants were used in this study. Merlin-UL128L^mut^ contains a premature stop codon mutation in UL128 that was selected during growth of Merlin in fibroblasts prior to BAC cloning. This mutation was repaired in Merlin-UL128L^wt^. In addition, because the FIX and TB40-BAC4 clones contain mutations in RL13 (38), which invariably mutates upon passage in fibroblast or epithelial cells (37, 38), a preexisting RL13 frameshift mutation present in these Merlin BACs was not repaired. TR contains an intact RL13, which could contribute to the growth characteristics of this virus. However, given the speed with which mutants are consistently selected in RL13 when clinical virus is grown *in vitro* (37, 38), it seems likely that RL13 is either not expressed or is nonfunctional in virus derived from TR.

Interestingly, all three protein-coding regions in UL128L appear to be intact in the TB40-BAC4, FIX, and TR BACs, yet reconstituted viruses have not been reported to acquire obvious mutations during passage in fibroblasts. It is possible that these three BACs contain natural variants of UL128L that are stable in fibroblasts, or that subtle UL128L mutations have been selected that preserve the integrity of the protein-coding regions but suppress their functions. Unfortunately, the clinical samples from which these strains were derived are not available for comparison. Therefore, we compared the growth properties of TB40-BAC4, FIX, and TR to those of Merlin-UL128L^wt^ and Merlin-UL128L^mut^. To monitor infection, all viruses were engineered to express EGFP from an IRES inserted downstream from UL122 (encoding IE2) (Table 1).

**Table 1:** BAC-cloned and recombinant HCMV strains used in this study

Strain	Reference	UL128L origin^*a*^	GenBank accession no.	Designation in text
BAC cloned				
Merlin^*b*^	38	Mutated: G>A in UL128 at nt 176260 (R>stop)	GU179001.1	Merlin-UL128L^mut^
Merlin^*b*^	38	Native	GU179001.1	Merlin-UL128L^wt^
TR^*b*^	51	Native	AC146906.1	TR
TB40-BAC4	48	Native	EF999921.1	TB40-BAC4
FIX^*b*^	50	Native	AC146907.1	FIX
Clinical sample (nonpassaged)				
3301	40	Native	GQ466044.1	3301
Recombinant Merlin containing variant UL128L				
Merlin		3301		Merlin-UL128L^3301^
Merlin		TR		Merlin-UL128L^TR^
Merlin		TB40-BAC4		Merlin-UL128L^TB40^
Merlin		FIX		Merlin-UL128L^FIX^
Recombinant Merlin strains containing unique substitutions in UL128L				
Merlin		TB40-BAC4: G>T in UL128 at nt 176663 (near splice acceptor site)		Merlin-UL128^G>T^
Merlin		FIX: A>G in UL130 at nt 177364 (S72P)		Merlin-UL130^A>G^

a Nucleotide positions are relative to the sequence of BAC-cloned HCMV strain Merlin (GU179001).

b GenBank accession numbers for parental viruses are NC_006273 (Merlin), GU179289 (VR1814; parental virus of FIX), and KF021605 (TR).

**Fig 1:**
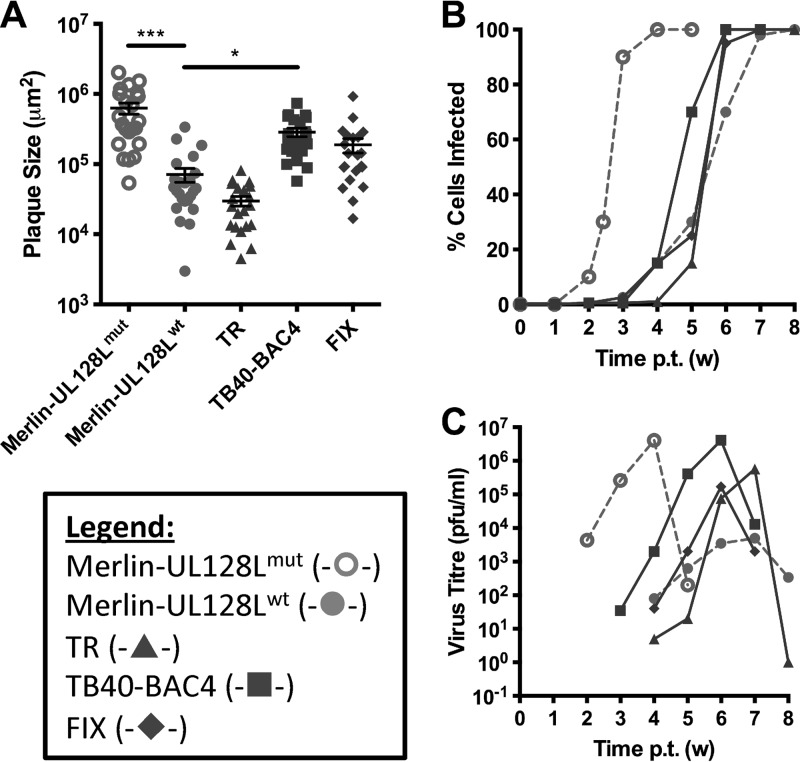
Growth characteristics of BAC-cloned strains in fibroblasts. (A) HFFF cells were transfected with BAC DNA for the indicated viruses and then placed under semisolid overlay. Plaque sizes were measured 2 weeks later. Means and standard deviations are shown. (B) HFFF cells were transfected with BAC DNA for the indicated viruses, and infection was allowed to progress until the monolayer was destroyed. At weekly time points posttransfection (p.t.), the level of infection was estimated by FACS analysis of EGFP-expressing cells. (C) Supernatants from the infections shown in panel B were retained at weekly intervals and titrated on HFFFs to provide a measure of cell-free virus release. (Where indicated, samples were compared by 1-way analysis of variance [ANOVA] followed by Dunnett's posttest to compare each sample to Merlin-UL128^wt^. *, *P* < 0.05; **, *P* < 0.01; ***, *P* < 0.001).

UL128L mutants can arise within a single passage of wild-type HCMV in fibroblasts (43). To ensure that the experiments were initiated using genetically homogenous virus preparations, HFFF cells were transfected with infectious BAC clones of each virus. The capacity of virus infection to progress by cell-to-cell spread was assessed by direct measurement of plaque sizes formed under a semisolid overlay (Fig. 1A). In this assay, Merlin-UL128L^mut^ consistently generated the largest plaques, whereas TB40-BAC4 and FIX plaques were smaller than those of Merlin-UL128L^mut^ but were 4- to 2.5-fold larger, respectively, than those of Merlin-UL128L^wt^. TR produced the smallest plaques of all viruses tested.

Consistent with previous work (38), Merlin-UL128L^mut^ spread through the HFFF monolayer the fastest (Fig. 1B) and produced the greatest amounts of cell-free virus (Fig. 1C), whereas Merlin-UL128L^wt^ spread the slowest and produced the lowest cell-free titers (approximately 1,000-fold less than Merlin-UL128L^mut^). Compared to Merlin-UL128L^wt^, TB40-BAC4 and FIX each spread through the HFFF monolayer faster and produced much higher yields (1,000- and 50-fold, respectively) of cell-free virus. Indeed, the amount of cell-free TB40-BAC4 release was similar to that of Merlin-UL128L^mut^. The TR infections spread very slowly at first, but the rate increased from week 5. Like TB40-BAC4 and FIX, TR also produced peak titers of cell-free virus that exceeded those of Merlin-UL128L^wt^ by more than 100-fold.

Thus, TB40-BAC4 and FIX displayed more efficient cell-to-cell spread in fibroblasts and produced greater yields of cell-free virus than Merlin containing wild-type UL128L. Cell-to-cell spread of TR in fibroblasts was reduced compared to that of Merlin containing wild-type UL128L, yet peak cell-free titers were significantly higher.

### Growth properties of HCMV strains FIX, Merlin, TB40-BAC4, and TR in epithelial cells.

**Fig 2:**
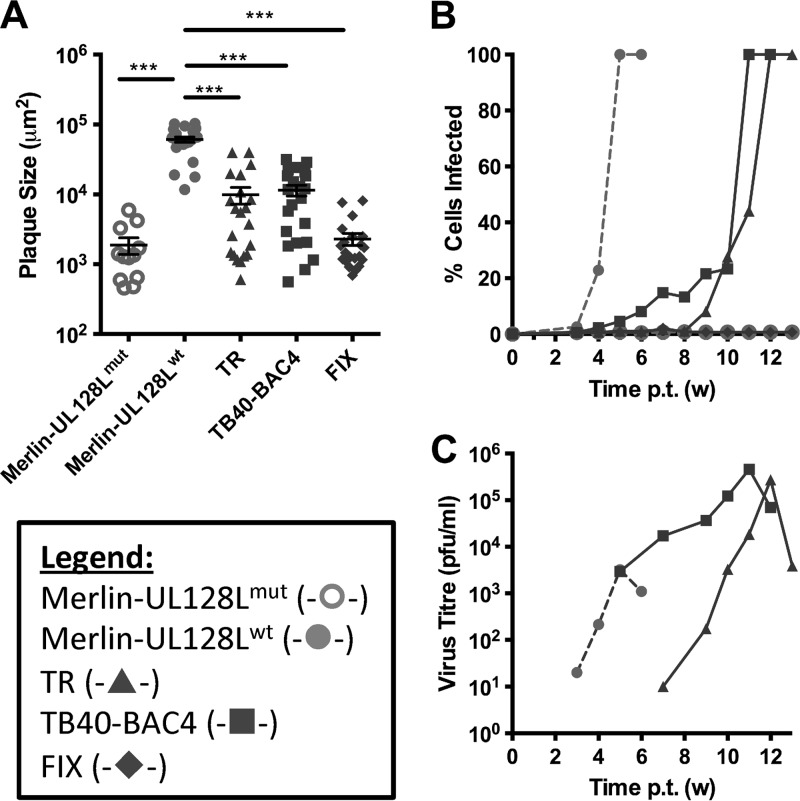
Growth characteristics of BAC-cloned strains in epithelial cells. (A) RPE-1 cells were transfected with BAC DNA for the indicated viruses and then placed under semisolid overlay. Plaque sizes were measured 3 weeks later. Means and standard deviations are shown. (B) RPE-1 cells were transfected with BAC DNA for the indicated viruses, and infection was allowed to progress until the monolayer was destroyed. At weekly time points, cells were trypsinized and the level of infection was measured by FACS analysis of EGFP-expressing cells. (C) Supernatants from the infections shown in panel B were retained at weekly intervals and titrated on HFFFs to provide a measure of cell-free virus release. (Where indicated, samples were compared by 1-way ANOVA followed by Dunnett's posttest to compare each sample to Merlin-UL128^wt^. *, *P* < 0.05; **, *P* < 0.01; ***, *P* < 0.001).

In order to investigate the growth characteristics of FIX, Merlin, TB40-BAC4, and TR in an epithelial cell line (which requires UL128L for efficient infection), RPE-1 cells were transfected with the infectious BAC clones. In marked contrast to the findings for fibroblasts, Merlin-UL128L^wt^ displayed much more efficient plaque formation than Merlin-UL128L^mut^ (Fig. 2A). TB40-BAC4 and TR plaques were of an intermediate size (approximately 6 times smaller than Merlin-UL128^wt^), while FIX formed plaques that were 25 times smaller, similar to those produced by Merlin-UL128L^mut^. TB40-BAC4 and TR each spread throughout the RPE-1 monolayer much more slowly than Merlin-UL128L^wt^ (Fig. 2B and C) yet produced 100- to 150-fold higher titers of cell-free virus. Neither FIX nor Merlin-UL128L^mut^ supported significant spread through RPE-1 monolayers.

Thus, the growth phenotypes displayed by TB40-BAC4, FIX, and TR in fibroblasts and epithelial cells were distinct from those of Merlin containing wild-type UL128L. Cell-to-cell spread of TB40-BAC4 and FIX was more efficient in fibroblasts but less efficient (along with TR) in epithelial cells. TB40-BAC4, TR, and FIX all produced significantly higher cell-free titers than Merlin-UL128L^wt^ in both cell types.

### The differential growth characteristics of TB40-BAC4 and FIX are determined by UL128L.

**Fig 3:**
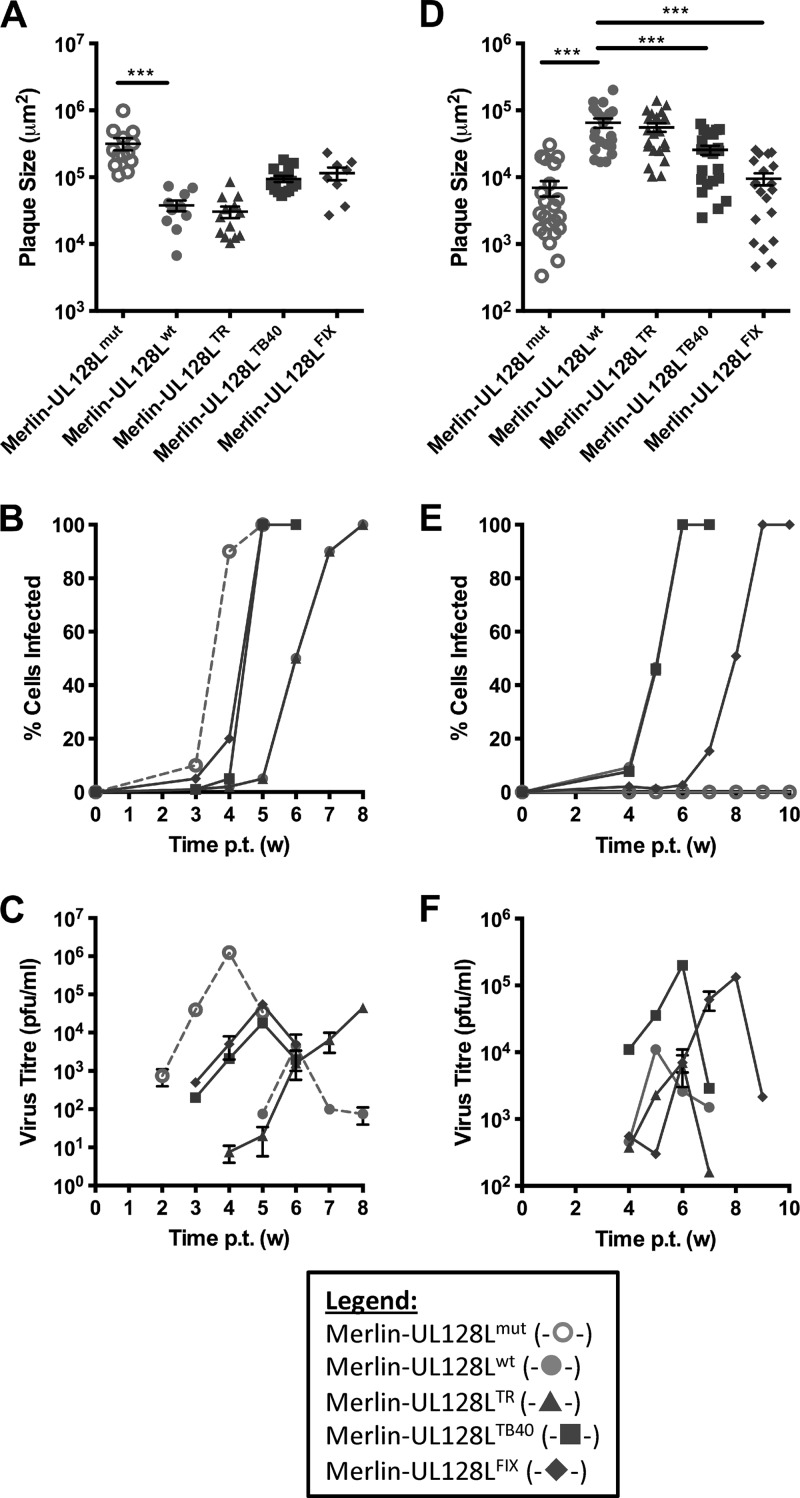
Growth characteristics of recombinant Merlin viruses containing UL128L from other strains. HFFF (A) or RPE-1 (D) cells were transfected with BAC DNA for the indicated viruses and then placed under semisolid overlay. Plaque sizes were measured 2 (A) or 3 (D) weeks later. Means and standard deviations are shown. HFFF (B) or RPE-1 (E) cells were transfected with BAC DNA for the indicated viruses, and infection was allowed to progress until the monolayer was destroyed. At weekly time points, the level of infection was estimated from EGFP expression (B), or cells were trypsinized and the level of infection was measured by FACS analysis of EGFP-expressing cells (E). (C and F) Supernatants from the infections shown in panels B and E were retained at weekly intervals and titrated on HFFFs to provide a measure of cell-free virus release. (Where indicated, samples were compared by 1-way ANOVA followed by Dunnett's posttest to compare each sample to Merlin-UL128^wt^. *, *P* < 0.05; **, *P* < 0.01; ***, *P* < 0.001.)

To assess whether the growth characteristics of each strain could be attributed to UL128L, we replaced UL128L in Merlin with that from TB40-BAC4, FIX, or TR, thereby generating the recombinant viruses Merlin-UL128L^TB40^, Merlin-UL128L^FIX^, and Merlin-UL128L^TR^, respectively (Table 1). Compared to Merlin-UL128L^wt^, acquisition of TB40-BAC4 or FIX UL128L was consistently associated with a 2.5- to 3-fold increased plaque size in fibroblasts (Fig. 3A), an increased rate of cell-free spread (Fig. 3B), and approximately 10-fold increased yields of cell-free virus (Fig. 3C). In contrast, TR UL128L did not alter the rate of cell-to-cell spread of strain Merlin and produced titers of cell-free virus that were comparable to those of Merlin-UL128L^wt^, at least until week 6 posttransfection. However, production of cell-free virus by Merlin-UL128L^TR^ increased dramatically by week 7. DNA sequencing of UL128L from cell-free virus during the final time point revealed that all viruses retained their original sequence in UL128L, with the exception of Merlin-UL128L^TR^. Merlin-UL128L^TR^ harbored a deletion (nt 176742 to 177247) affecting both UL130 and UL128, as well as a G-to-A mutation at nt 177355 that resulted in a premature stop codon in UL130. Thus, the rapid increase in cell-free virus production by Merlin-UL128L^TR^ was correlated with mutation of UL128L.

Measurement of the size of plaques formed by these recombinant viruses in RPE-1 cells (Fig. 3D) revealed that expression of TB40-BAC4 and FIX UL128L reduced the epithelial cell-to-cell spread of Merlin, with plaques 2.5 to 7 times smaller, respectively. However, TR UL128L did not affect the growth characteristics of Merlin. Likewise, time course experiments in epithelial cells showed that transfer of FIX UL128L resulted in reduced speed of dissemination, and transfer of FIX and TB40-BAC4, but not TR, UL128L resulted in 10- to 20-fold increased production of cell-free virus (Fig. 3E and F). Thus, compared to Merlin-UL128L^wt^, the ability of TB40-BAC4 and FIX to produce larger plaques in fibroblasts but smaller plaques in epithelial cells, as well as to produce higher titers of cell-free virus in both cell types, appears to be attributable at least in part to genetic differences in UL128L. However, the differential growth characteristics between TR and Merlin-UL128L^wt^ appear to be independent of UL128L.

### A single-nucleotide difference in a UL128 intron increases cell-free virus production.

TB40-BAC4 infection produced significantly higher titers of cell-free virus than Merlin-UL128L^wt^ in both epithelial and fibroblast cells. Since UL128L clearly makes a major contribution to the differential growth characteristics of TB40-BAC4, we hypothesized that UL128L had acquired a mutation during passage that is compatible with growth in fibroblasts yet permits the virus to retain a degree of epithelial cell tropism. If this were the case, any such mutation would be expected to be unique to TB40-BAC4. UL128L sequences from different strains exhibit a high level of sequence conservation (>92.3% at the nucleotide level [64, 65]); however, BLAST searches revealed that TB40-BAC4 had a unique G-to-T (G>T) substitution at nt 176612 (with reference to the equivalent location in the Merlin BAC genome). The G residue is conserved in 50 other HCMV strains and is located in the first of the two UL128 introns, 6 nt from the splice acceptor site. To determine whether this substitution affects the growth properties of TB40-BAC4, it was introduced into Merlin-UL128L^wt^ to generate Merlin-UL128^G>T^ (Table 1). As an additional control, UL128L from strain 3301 was inserted into Merlin (generating Merlin-UL128L^3301^). Strain 3301 had not been subjected to *in vitro* passage prior to sequencing and was thus representative of a UL128L sequence that was different from that of Merlin but that was known to be wild type. UL128L from 3301 was also highly homologous to UL128L from TB40-BAC4 (99.49% at the nucleotide level); however, it did not contain the G>T nucleotide difference identified in the intron of UL128. Therefore, it was useful to determine whether the G>T substitution, as opposed to natural strain variation elsewhere, contributed to the different characteristics of UL128L from TB40-BAC4 compared to that from Merlin.

**Fig 4:**
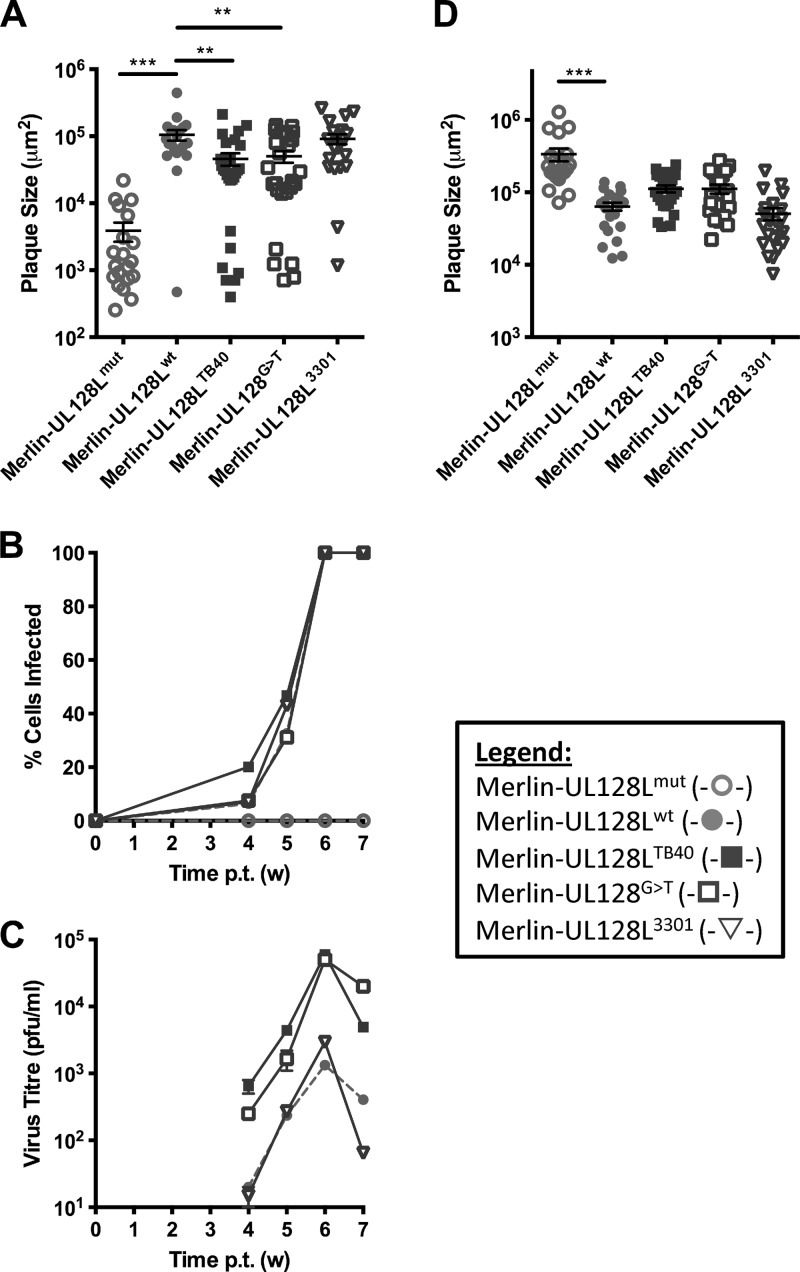
Characteristics of virus containing TB40-BAC4 UL128 G>T unique nucleotide. (A) RPE-1 cells were transfected with BAC DNA for the indicated viruses and then incubated under semisolid overlay. Three weeks later plaque sizes were measured. Means and standard deviations are shown. (B) RPE-1 cells were transfected with BAC DNA for the indicated viruses, and infection was allowed to progress until the monolayer was destroyed. At weekly time points, cells were trypsinized and the level of infection was measured using FACS analysis of EGFP-expressing cells. (C) Also at weekly time points, supernatants were kept and titrated on HFFFs to provide a measure of cell-free virus release. (D) HFFF cells were transfected with BAC DNA for the indicated viruses and then placed under semisolid overlay. Two weeks later, plaque sizes were measured. Means and standard deviations are shown. (Where indicated, samples were compared by 1-way ANOVA followed by Dunnett's posttest to compare each sample to Merlin-UL128^wt^. *, *P* < 0.05; **, *P* < 0.01; ***, *P* < 0.001.)

Merlin-UL128L^wt^ and Merlin-UL128L^3301^ displayed similar cell-cell spread properties in RPE-1 cells. Transfer of the substitution identified in TB40-BAC4 UL128 was sufficient to reduce epithelial cell-to-cell spread of Merlin to the same degree (approximately 2-fold) as transfer of the entire TB40-BAC4 UL128L (Fig. 4A). Investigations of infection kinetics (Fig. 4B) and production of cell-free virus in epithelial cell culture (Fig. 4C) also showed that Merlin-UL128L^3301^ had growth characteristics similar to those of Merlin-UL128L^wt^. Transfer of the G>T substitution to Merlin did not alter the rate of spread through the monolayer, but it did increase the production of cell-free virus by Merlin by the same degree as transfer of the entire TB40-BAC4 UL128L (approximately 50-fold). When plaque sizes were measured in fibroblasts (Fig. 4D), Merlin-UL128L^3301^ again formed plaques of sizes comparable to those of Merlin-UL128L^wt^, whereas the plaques formed by Merlin-UL128^G>T^ were similar in size to those formed by Merlin-UL128L^TB40^ and were consistently 2-fold larger than those formed by Merlin-UL128L^wt^ and Merlin-UL128L^3301^.

### A single-nucleotide difference in a TB40 UL128 intron reduces splicing efficiency.

**Fig 5:**
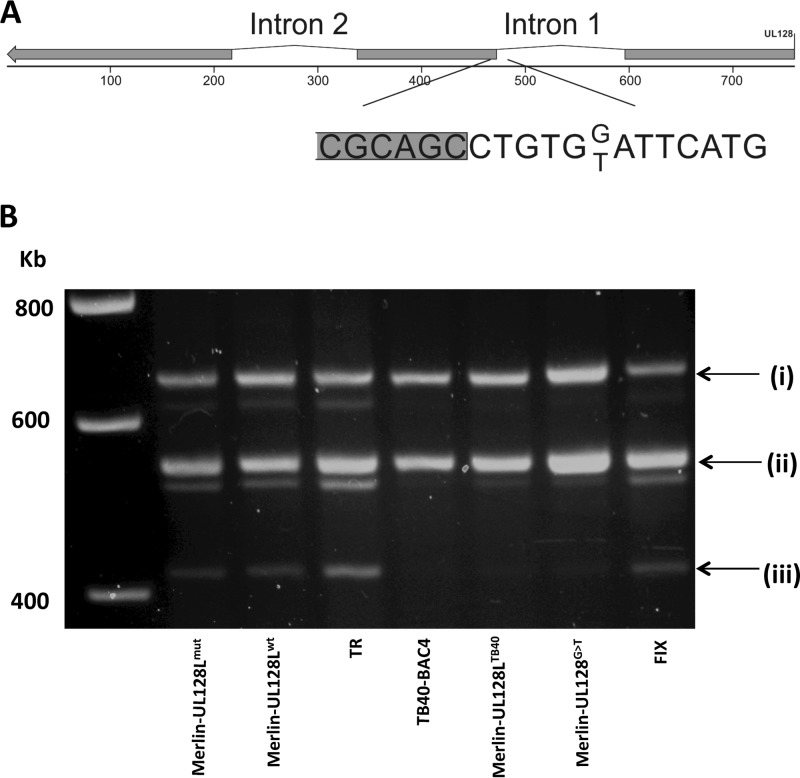
UL128 gene map and transcripts. (A) The position of the unique A residue identified in TB40-BAC4 UL128 intron 1, in place of the C residue present in all other strains, is indicated. Protein-coding exons are shaded. (B) HFFF cells were infected with the indicated virus, and total RNA was extracted at 72 h postinfection. RT-PCR was performed using primers binding in the first and third exons of UL128. The bands correspond to transcripts that are (i) unspliced, (ii) lacking intron 2, or (iii) lacking both intron 1 and intron 2.

The proximity of the G>T substitution in TB40-BAC4 UL128 to the splice acceptor site in intron 1 suggested that it had the potential to disrupt splicing of UL128 mRNA. Indeed, bioinformatics analysis predicted that this would be the case (Fig. 5A). Total infected cell RNA was extracted 72 h postinfection, and RT-PCR was performed across both introns of UL128 (Fig. 5B). Three differently spliced products were detected, and DNA sequencing demonstrated that they corresponded to (i) an unspliced transcript (656 to 662 bp, depending on the strain), (ii) a transcript with intron 2 excised (540 bp), and (iii) a transcript with both introns 1 and 2 excised (417 bp). All three UL128 products were detected in all strains tested; however, in the constructs containing the G>T substitution (TB40-BAC4, Merlin-UL128L^TB40^, and Merlin-UL128^G>T^), the cDNAs corresponding to the fully spliced transcript were present at significantly lower abundance.

Thus, the G>T substitution identified in TB40-BAC4 reduced the efficiency of UL128 mRNA splicing and appeared to be entirely responsible for the different growth characteristics conferred by TB40-BAC4 UL128L compared to those of Merlin UL128L.

### A single-nucleotide difference in FIX UL130 increases virus production.

**Fig 6:**
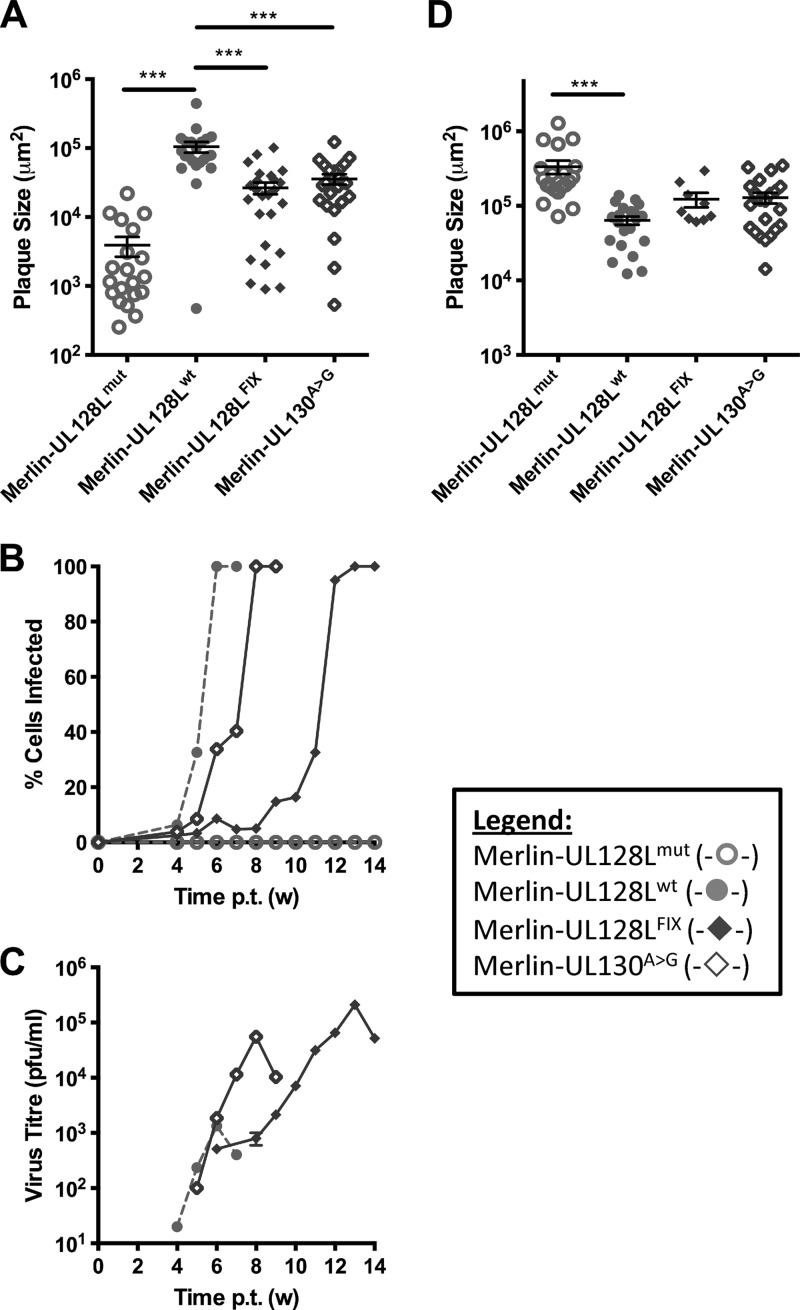
Growth characteristics of virus containing the FIX UL130 A>G substitution. (A) RPE-1 cells were transfected with BAC DNA for the indicated viruses and incubated under semisolid overlay. Plaque sizes were measured 3 weeks later. Means and standard deviations are shown. (B) RPE-1 cells were transfected with BAC DNA for the indicated viruses, and infection was allowed to progress until the monolayer was destroyed. At weekly time points, cells were trypsinized and the level of infection was measured by FACS analysis of EGFP-expressing cells. (C) Supernatants from the infections shown in panel B were retained at weekly intervals and titrated on HFFFs to provide a measure of cell-free virus release. (D) HFFF cells were transfected with BAC DNA for the indicated viruses and then placed under semisolid overlay. Plaque sizes were measured 2 weeks later. Means and standard deviations are shown. (Where indicated, samples were compared by 1-way ANOVA followed by Dunnett's posttest to compare each sample to Merlin-UL128^wt^. *, *P* < 0.05; **, *P* < 0.01; ***, *P* < 0.001.)

BLAST searches of UL128L sequences also revealed a unique A-to-G (A>G) substitution in FIX UL130 at nt 177364 (with reference to the Merlin BAC sequence). This difference manifests as a serine-to-proline amino acid change (S72P), and according to protein structure prediction, it disrupts a beta sheet within the protein. The alteration was introduced into Merlin-UL128L^wt^ (generating Merlin-UL130^A>G^) (Table 1). Merlin-UL130^A>G^ produced plaques in RPE-1 cells approximately 3-fold smaller than those of Merlin-UL128L^wt^ but similar in size to those formed by Merlin-UL128L^FIX^ (Fig. 6A). The effects of this substitution on dissemination during infection (Fig. 6B) and cell-free virus production (Fig. 6C) in epithelial cell culture were also investigated. It reduced the rate of dissemination through the epithelial cell monolayer compared to that of Merlin-UL128L^wt^, although not as much as transfer of the entire FIX UL128L. It also increased the production of cell-free virus approximately 50-fold compared to that of Merlin-UL128L^wt^. As with the rate of spread through the monolayer, the increase in cell-free release was not as dramatic as that achieved by transfer of the entire FIX UL128L (approximately 150-fold). When cell-to-cell spread ability was tested in HFFFs (Fig. 6D), Merlin-UL130^A>G^ and Merlin-UL128L^FIX^ formed plaques of comparable size, and the plaques were consistently twice as large as those formed by Merlin-UL128L^wt^.

Thus, the A>G substitution in FIX UL130 contributed significantly to the different growth characteristics of FIX UL128L compared to those of Merlin UL128L.

### pUL128 is reduced in TB40-BAC4 and FIX virions.

**Fig 7:**
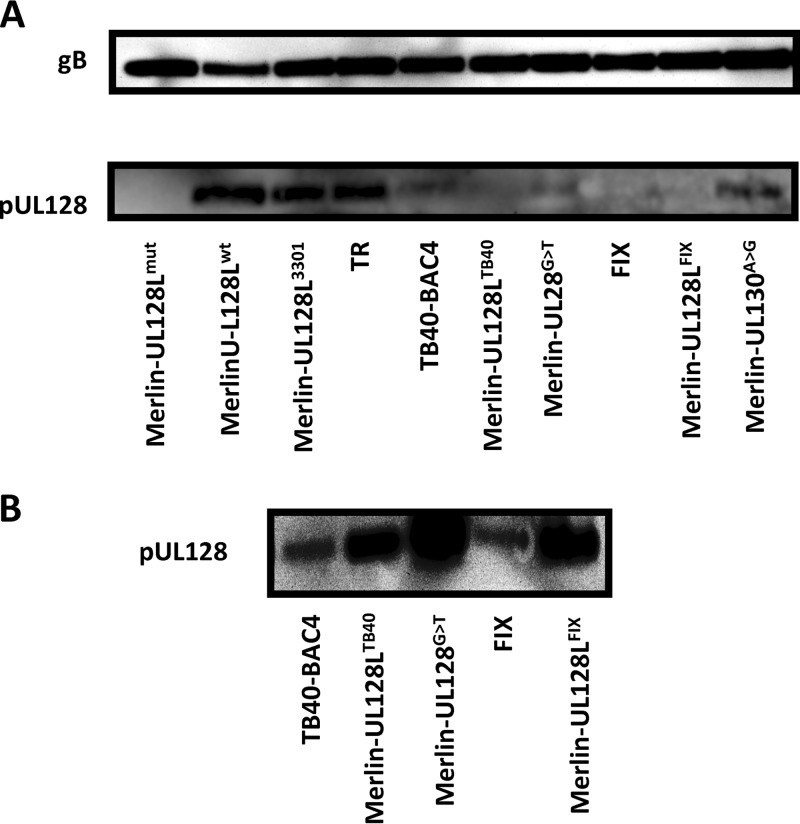
Virion pUL128 content of BAC-cloned strains and recombinant Merlin viruses. (A) Stocks of the indicated viruses were grown, and infectious virions were purified on glycerol-tartrate gradients before being analyzed by Western blotting. Loading was normalized to gB levels (upper panel) and stained for pUL128 (lower panel). (B) The same samples as those used for panel A were analyzed by Western blotting. However, rather than being normalized to gB content, virion loads were maximized to facilitate detection of pUL128 in each strain.

The substitutions in TB40-BAC4 and FIX UL128L identified above have the potential to influence the function of gH/gL/UL128L by various mechanisms. Impairing the efficiency of RNA splicing in TB40-BAC4 would be expected to limit expression of full-length pUL128, and the S72P substitution in pUL130 might reduce the stable incorporation of pUL130 into gH/gL/UL128L or affect a functional domain of the complex. Since all five subunits are required for stable incorporation of gH/gL/UL128L into the virion (28, 66), alteration of the amounts or structural attributes of any one component has the potential to affect the levels of the entire complex incorporated into the virion. To investigate this, levels of pUL128 were analyzed in HCMV virions purified on glycerol-tartrate gradients (Fig. 7A), with sample loading normalized to that of gB. Note that although the epitope recognized by the anti-UL128 antibody used in this assay is not mapped, any differences in detection are unlikely to be due to different antibody affinities for pUL128 from different strains; compared to Merlin, UL128 in the other strains differed by only one (FIX) or two (TB40-BAC4 and TR) amino acids, while in Merlin viruses containing the single-nucleotide substitutions from FIX or TB40-BAC4, pUL128 is identical to Merlin.

pUL128 was undetectable in Merlin-UL128L^mut^ but was readily detected in viruses having wild-type UL128L sequences (Merlin-UL128L^wt^ and Merlin-UL128L^3301^). Consistent with TR UL128L having a similar influence on growth kinetics compared to Merlin UL128L, TR contained levels of pUL128 that were comparable to those in Merlin-UL128L^wt^ and Merlin-UL128L^3301^. However, TB40-BAC4 contained reduced levels of pUL128, as did Merlin containing either the entire TB40-BAC4 UL128L region (Merlin-UL128L^TB40^) or only the G>T substitution (Merlin-UL128^G>T^). pUL128 was undetectable in FIX and Merlin-UL128L^FIX^, while Merlin-UL130^A>G^ contained reduced, though detectable, levels of pUL128.

Based on the spread of these viruses in epithelial cells, it seemed likely that all except Merlin-UL128L^mut^ contained gH/gL/UL128L in the virion, and that lack of detection of pUL128 in some was due to the small amounts of virus loaded in order to keep sample loading comparable to that of the low-titer viruses Merlin-UL128L^wt^ and Merlin-UL128L^3301^. In a separate blot where the virion load was not normalized, pUL128L was detectable in all virions except Merlin-UL128L^mut^ (Fig. 7B).

Thus, all virions except Merlin-UL128^mut^ contain pUL128; however, the substitutions identified in UL128L of TB40-BAC4 and FIX resulted in reduced levels of gH/gL/UL128L being incorporated into the virion.

### TB40-BAC4 and FIX UL128L bestow impaired epithelial cell tropism.

**Fig 8:**
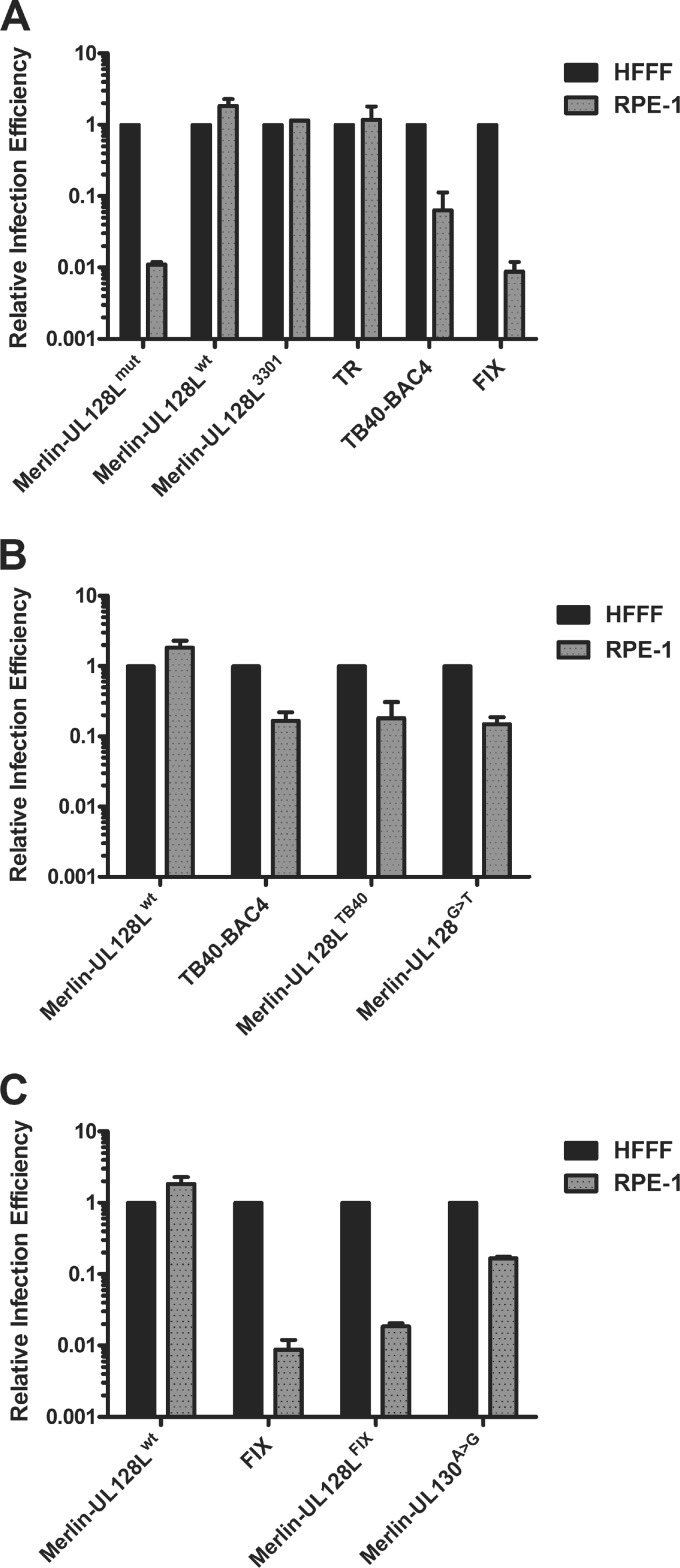
Relative infection efficiency of fibroblast and epithelial cells. Stocks of the indicated viruses were simultaneously titrated onto HFFF or RPE-1 cells. Titers for each virus on RPE-1 cells were then normalized to the titer of the same virus on HFFF cells, which was arbitrarily set to 1, giving the relative efficiency of infection of epithelial cells for each strain. The data are based on two independent experiments. (A) Relative infection efficiencies of TR, TB40-BAC4, and FIX compared to various Merlin viruses. (B) Relative infection efficiencies of Merlin containing either the entire UL128L or the substitution from TB40-BAC4 compared to TB40-BAC4 and Merlin. (C) Relative infection efficiencies of Merlin containing either the entire UL128L or the single nucleotide from FIX compared to FIX and Merlin.

Since gH/gL/UL128L is required for efficient infection of epithelial cells, its reduced incorporation into virions would be expected to restrict epithelial cell tropism. Viruses derived from each BAC-cloned strain were plaque titrated on HFFF and RPE-1 cells in parallel (Fig. 8A). For each virus, the titer reported in RPE-1 cells was normalized to that reported for HFFF cells for the same virus, thereby quantitating the ability of each virus to infect epithelial cells relative to its ability to infect fibroblasts. Merlin-UL128L^mut^ infected RPE-1 cells approximately 100-fold less efficiently than HFFFs; however, the majority of plaques were represented by single cells, indicating that cell-to-cell spread was strongly inhibited. Viruses containing wild-type UL128L (Merlin-UL128L^wt^ and Merlin-UL128L^3301^) infected RPE-1 cells with comparable or slightly greater efficiency than they did HFFFs. Like Merlin-UL128L^wt^ and Merlin-UL128L^3301^, TR infected RPE-1 and HFFF cells with similar efficiencies, consistent with these viruses containing comparable levels of gH/gL/UL128L in virions. However, infection of RPE-1 cells by TB40-BAC4 was approximately 10-fold less efficient than infection of HFFFs, whereas the efficiency of infection of RPE-1 cells by FIX was approximately 100-fold less than that of HFFFs and comparable to a virus lacking gH/gL/UL128L (Merlin-UL128L^mut^).

Like TB40-BAC4, Merlin-UL128L^TB40^ and Merlin-UL128^G>T^ infected RPE-1 cells approximately 10-fold less efficiently than HFFFs (Fig. 8B), displaying a lower infection efficiency than Merlin-UL128L^wt^, Merlin-UL128L^3301^, and TR.

FIX infected epithelial cells with approximately 100-fold less efficiency than fibroblasts, and this was closely recapitulated by Merlin containing FIX UL128L (Fig. 8C). The A>G substitution in FIX UL130 also reduced the ability of Merlin to infect RPE-1 cells, although only by approximately 10-fold.

In summary, the unique nucleotide differences identified in TB40-BAC4 UL128 and FIX UL130 resulted in a reduction in the amounts of gH/gL/UL128L incorporated into the virion and a concomitant reduction in the relative ability of virus to infect epithelial cells.

## DISCUSSION

Previous attempts to propagate wild-type HCMV from clinical material by passage *in vitro* in fibroblasts have shown that mutations invariably occur in the viral genome. RL13 mutates first, followed by UL128L, and following these steps virus grows to much higher cell-free titers (37, 38). However, the adapted virus lacks gH/gL/UL128L in the virion envelope; therefore, it does not efficiently infect cells other than fibroblasts. The single-nucleotide substitutions identified in UL128L of TB40-BAC4 and FIX may explain the apparently conflicting fact that these strains retain the ability to infect endothelial and epithelial cells when passaged on fibroblasts. gH/gL/UL128L is expressed and is present in virions of these strains, but the level is reduced. This permits greater release of cell-free virus and potentially reduces the selective pressure for further mutations in UL128L.

It is important to determine whether these substitutions represent natural strain variation or mutations acquired *in vitro*. The clinical material from which the strains were derived is not available; therefore, it is not possible to answer this question directly. However, the S72P substitution in FIX UL130 is absent from the passaged parental strain VR1814 (37); thus, it probably occurred during further passage of VR1814 prior to BAC cloning. Similarly, the fact that the G>T substitution in intron 1 of TB40-BAC4 is unique to TB40-BAC4 strongly suggests that it was acquired during passage in fibroblasts prior to BAC cloning. This mutation is also absent from the consensus sequence of the parental strain (TB40/E), which instead contains a unique C207S variation in UL130. TB40/E is known to comprise a mixture of genomes (48), and the G>T mutation may be present at low levels in this mixture. Nevertheless, the identification of the substitutions in FIX and TB40-BAC4 demonstrates the importance of comparing viral genomes to unpassaged virus when determining whether a gene is wild type in sequence and whether (by extension) the phenotype of a virus can be assumed to be the same as the clinical virus.

The effect of these substitutions was a reduction in levels of gH/gL/UL128L in the virion. This manifested in impaired efficiency of epithelial cell entry, an increase in cell-to-cell spread in fibroblasts but a decrease in cell-to-cell spread in epithelial cells, and an increase in cell-free release in both cell types. Taken together, these observations indicate that the level of gH/gL/UL128L in the virion is directly related to its ability to inhibit replication in fibroblasts and to promote entry and cell-to-cell spread in epithelial cells. Surprisingly, it also suggests that wild-type UL128L inhibits the production of cell-free virus in both epithelial and fibroblast cells. The mechanism by which this occurs is unclear but may be related to the fact that gH and gL are common to two glycoprotein complexes (gH/gL/UL128L and gH/gL/gO). Greater amounts of gH/gL/UL128L may result in there being less gH/gL available for the formation of gH/gL/gO. gH/gL/gO may be important for secondary envelopment of progeny virions and egress into the supernatant (23) and for subsequent infection by cell-free released virus (24, 67), explaining the increased titers of cell-free virus as the level of gH/gL/UL128L is reduced. This model is supported by the observation that expression of wild-type UL128L reduces levels of both cell-free and cell-associated virus (37), and that loss of gO results in greater accumulation of gH/gL/UL128L in the virion with a concomitant increase in cell-to-cell spread in epithelial and endothelial cells (24).

Although the mutations identified in FIX and TB40-BAC4 strongly contributed to the greater production of cell-free virus and differences in cell-to-cell spread, it is clear that they are not the sole reason for the growth differences seen in TB40-BAC4 and FIX compared to Merlin-UL128L^wt^. TB40-BAC4 grew to approximately 1,000-fold higher cell-free titers than Merlin-UL128L^wt^, whereas transfer of the G>T mutation in TB40-BAC4 UL128 resulted in an increase of only 50-fold. Likewise, FIX was unable to spread cell to cell in RPE-1 epithelial cells, yet Merlin-UL128L^FIX^ was able to spread, albeit more slowly than Merlin-UL130^A>G^, which itself spread more slowly than Merlin-UL128L^wt^. Similar considerations apply to TR. Despite containing levels of gH/gL/UL128L similar to those in Merlin-UL128L^wt^, TR generated higher cell-free titers yet displayed lower efficiency of cell-to-cell spread in both fibroblast and epithelial cells. The observation that Merlin-UL128L^TR^ behaved the same as Merlin-UL128L^wt^ and Merlin-UL128^3301^, and that TR UL128L readily mutated when expressed in the Merlin genome during growth in fibroblasts but not when expressed within the TR genome, underlines the conclusion that the differential growth characteristics of TR were independent of UL128L yet are sufficient to enhance the stability of UL128L *in vitro*. These differences may be due to a number of other genome regions that can influence growth in a cell type-specific manner (37, 68–70). Sequence differences in other glycoproteins might also be responsible, with gO being a particularly prominent candidate because it exists in several highly divergent, yet stable, genotypes (43, 71–73). Alignments of the protein-coding regions of gH, gL, gM, gN, gO, and gB of TR, TB40-BAC4, and FIX with those of other strains did not reveal any unique substitutions that could represent *in vitro* adaptations, although all 3 viruses lack sequences in Us, where the BAC cassette was inserted, and TB40-BAC4 contains mutations in RL5A, RL6, UL141, and UL40 (48, 74), and TR contains a mutation in UL97 (C607Y) that conveys ganciclovir resistance (75). These mutations could also contribute to the growth characteristics observed.

Owing to its central role in determining tropism for a broad range of clinically significant cell types, it is essential that an intact, wild-type gH/gL/UL128L is present in any strain used to investigate HCMV pathogenesis. More recently, other important implications of UL128L function have underscored the need for research based on virus strains that are competent in this genome region. Specifically, gH/gL/UL128L elicits a potent neutralizing antibody response in a natural infection (76–81) and is considered ideal for inclusion in new vaccine strategies (34, 82). The differences identified between levels of gH/gL/UL128L in various strains analyzed have the potential to affect read-outs of the efficacy of anti-gH/gL/UL128L antibodies. However, they also offer significant advantages relating to the important issue of being able to grow HCMV while minimizing the risk of mutation. For work performed in fibroblast cells, viruses based on Merlin-UL128L^mut^ offer an ideal defined, full-length genome that produces relatively high titers. For work requiring infection of other cell types, Merlin-UL128^G>T^ produces larger amounts of cell-free virus that is still able to infect epithelial and endothelial cells, potentially with reduced risk of mutation in UL128L.
